# 4-Ethyl-1-[4-(methyl­sulfan­yl)benzyl­idene]thio­semicarbazide

**DOI:** 10.1107/S1600536810022920

**Published:** 2010-06-18

**Authors:** Yu-Feng Li, Fang-Fang Jian

**Affiliations:** a Microscale Science Institute, Department of Chemistry and Chemical Engineering, Weifang University, Weifang 261061, People’s Republic of China; b Microscale Science Institute, Weifang University, Weifang 261061, People’s Republic of China

## Abstract

There are four independent mol­ecules in the asymmetric unit of the title compound, C_11_H_15_N_3_S_2_, with different conformations: the dihedral angles between the benzene rings and thio­urea units are 16.85 (9), 0.56 (10), 8.05 (12) and 4.56 (8)°. Each mol­ecule contains an intra­molecular N—H⋯N hydrogen bond, generating an *S*(5) ring. The crystal structure is stabilized by inter­molecular N—H⋯S hydrogen bonds.

## Related literature

For a related structure and background references to thio­semicarbazones, see: Li & Jian (2010 ▶).
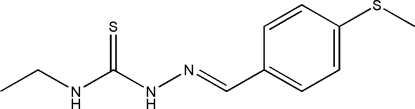


## Experimental

### 

#### Crystal data

C_11_H_15_N_3_S_2_
*M* *_r_* = 253.38
Triclinic, 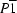

*a* = 10.496 (2) Å
*b* = 15.737 (3) Å
*c* = 17.542 (4) Å
α = 111.07 (3)°
β = 91.62 (3)°
γ = 100.43 (3)°
*V* = 2645.4 (9) Å^3^
*Z* = 8
Mo *K*α radiation
μ = 0.38 mm^−1^
*T* = 293 K
0.22 × 0.20 × 0.18 mm

#### Data collection

Bruker SMART CCD diffractometer
26033 measured reflections
12032 independent reflections
8042 reflections with *I* > 2σ(*I*)
*R*_int_ = 0.042

#### Refinement

*R*[*F* ^2^ > 2σ(*F* ^2^)] = 0.053
*wR*(*F* ^2^) = 0.201
*S* = 1.31
12032 reflections
577 parameters
H-atom parameters constrained
Δρ_max_ = 0.68 e Å^−3^
Δρ_min_ = −0.44 e Å^−3^

### 

Data collection: *SMART* (Bruker, 1997 ▶); cell refinement: *SAINT* (Bruker, 1997 ▶); data reduction: *SAINT*; program(s) used to solve structure: *SHELXS97* (Sheldrick, 2008 ▶); program(s) used to refine structure: *SHELXL97* (Sheldrick, 2008 ▶); molecular graphics: *SHELXTL* (Sheldrick, 2008 ▶); software used to prepare material for publication: *SHELXTL*.

## Supplementary Material

Crystal structure: contains datablocks global, I. DOI: 10.1107/S1600536810022920/hb5494sup1.cif

Structure factors: contains datablocks I. DOI: 10.1107/S1600536810022920/hb5494Isup2.hkl

Additional supplementary materials:  crystallographic information; 3D view; checkCIF report

## Figures and Tables

**Table 1:** Hydrogen-bond geometry (Å, °)

*D*—H⋯*A*	*D*—H	H⋯*A*	*D*⋯*A*	*D*—H⋯*A*
N1*A*—H1*AA*⋯N2*A*	0.86	2.20	2.602 (3)	108
N1*B*—H1*BA*⋯N3*B*	0.86	2.17	2.585 (3)	109
N1*C*—H1*CA*⋯N3*C*	0.86	2.23	2.624 (3)	108
N3*D*—H3*DA*⋯N1*D*	0.86	2.22	2.610 (3)	107
N3*A*—H3*AA*⋯S1*D*^i^	0.86	2.59	3.398 (2)	156
N2*D*—H2*DA*⋯S2*A*^ii^	0.86	2.57	3.402 (2)	163
N1*A*—H1*AA*⋯S1*B*	0.86	2.81	3.4798 (19)	136
N2*B*—H2*BA*⋯S1*C*	0.86	2.72	3.579 (2)	174
N2*C*—H2*CA*⋯S1*B*	0.86	2.64	3.487 (3)	168

Symmetry codes: (i) 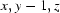; (ii) 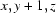.

**Fig. 1.**
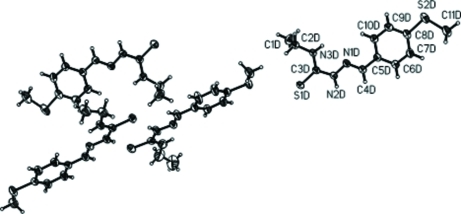
The structure of (I) showing 30% probability displacement ellipsoids.

## supplementary crystallographic information

### Comment

As part of our ongoing studies of thiosemicarbazone compounds (Li & Jian, 2010), we synthesized the title compound (I), and describe its structure here. In the four independent molecules, the dihedral angle between the benzene ring and the thiourea unit is [16.85 (9)°], [0.56 (10)°], [8.05 (12)°], [4.56 (8)°] respectively.

### Experimental

A mixture of 4-ethylthiosemicarbazide (0.1 mol), and 4-(methylthio)benzaldehyde (0.1 mol) was stirred in refluxing ethanol (20 ml) for 2 h to afford the title compound (0.086 mol, yield 86%). Colourless blocks of (I) were obtained by recrystallization from ethanol at room temperature.

### Refinement

H atoms were fixed geometrically and allowed to ride on their attached atoms, with C—H distances=0.97 Å, and with *U*_iso_=1.2–1.5*U*_eq_.

### Crystal data

**Table:**

C_11_H_15_N_3_S_2_	*Z* = 8
*M**_r_* = 253.38	*F*(000) = 1072
Triclinic, *P*1	*D*_x_ = 1.272 Mg m^−^^3^
Hall symbol: -P 1	Mo *K*α radiation, λ = 0.71073 Å
*a* = 10.496 (2) Å	Cell parameters from 8042 reflections
*b* = 15.737 (3) Å	θ = 3.0–27.5°
*c* = 17.542 (4) Å	µ = 0.38 mm^−^^1^
α = 111.07 (3)°	*T* = 293 K
β = 91.62 (3)°	Block, colorless
γ = 100.43 (3)°	0.22 × 0.20 × 0.18 mm
*V* = 2645.4 (9) Å^3^	

### Data collection

**Table:**

Bruker SMART CCD diffractometer	8042 reflections with *I* > 2σ(*I*)
Radiation source: fine-focus sealed tube	*R*_int_ = 0.042
graphite	θ_max_ = 27.5°, θ_min_ = 3.0°
phi and ω scans	*h* = −13→13
26033 measured reflections	*k* = −20→20
12032 independent reflections	*l* = −22→22

### Refinement

**Table:**

Refinement on *F*^2^	Primary atom site location: structure-invariant direct methods
Least-squares matrix: full	Secondary atom site location: difference Fourier map
*R*[*F*^2^ > 2σ(*F*^2^)] = 0.053	Hydrogen site location: inferred from neighbouring sites
*wR*(*F*^2^) = 0.201	H-atom parameters constrained
*S* = 1.31	*w* = 1/[σ^2^(*F*_o_^2^) + (0.1*P*)^2^] where *P* = (*F*_o_^2^ + 2*F*_c_^2^)/3
12032 reflections	(Δ/σ)_max_ = 0.001
577 parameters	Δρ_max_ = 0.68 e Å^−^^3^
0 restraints	Δρ_min_ = −0.44 e Å^−^^3^

### Special details

**Table:**

Geometry. All e.s.d.'s (except the e.s.d. in the dihedral angle between two l.s. planes) are estimated using the full covariance matrix. The cell e.s.d.'s are taken into account individually in the estimation of e.s.d.'s in distances, angles and torsion angles; correlations between e.s.d.'s in cell parameters are only used when they are defined by crystal symmetry. An approximate (isotropic) treatment of cell e.s.d.'s is used for estimating e.s.d.'s involving l.s. planes.
Refinement. Refinement of *F*^2^ against ALL reflections. The weighted *R*-factor *wR* and goodness of fit *S* are based on *F*^2^, conventional *R*-factors *R* are based on *F*, with *F* set to zero for negative *F*^2^. The threshold expression of *F*^2^ > σ(*F*^2^) is used only for calculating *R*-factors(gt) *etc*. and is not relevant to the choice of reflections for refinement. *R*-factors based on *F*^2^ are statistically about twice as large as those based on *F*, and *R*- factors based on ALL data will be even larger.

### Fractional atomic coordinates and isotropic or equivalent isotropic displacement parameters (Å^2^)

**Table:**

	*x*	*y*	*z*	*U*_iso_*/*U*_eq_	
S1D	0.70888 (7)	0.63167 (4)	0.27855 (3)	0.06536 (19)	
S2A	0.77477 (8)	−0.09229 (4)	0.25247 (3)	0.0713 (2)	
S1B	0.81356 (8)	−0.20823 (4)	−0.11837 (3)	0.0747 (2)	
S2D	0.91253 (8)	1.22890 (5)	0.82918 (4)	0.0787 (2)	
S1C	0.59612 (10)	−0.26828 (5)	−0.35997 (4)	0.0909 (3)	
S2C	0.57360 (9)	0.35118 (5)	0.15668 (5)	0.0892 (3)	
S1A	0.63732 (9)	−0.69992 (6)	−0.29039 (5)	0.0952 (3)	
S2B	1.00646 (10)	−0.79759 (6)	−0.60067 (5)	0.0999 (3)	
N3A	0.72112 (19)	−0.26196 (13)	0.14045 (10)	0.0570 (5)	
H3AA	0.6975	−0.2804	0.1795	0.068*	
N3D	0.6760 (2)	0.68551 (12)	0.43757 (10)	0.0601 (5)	
H3DA	0.6771	0.7297	0.4842	0.072*	
N2A	0.71480 (17)	−0.32505 (12)	0.06176 (10)	0.0522 (4)	
N2D	0.75067 (19)	0.80099 (12)	0.39072 (10)	0.0562 (4)	
H2DA	0.7700	0.8205	0.3517	0.067*	
N1D	0.76168 (17)	0.86248 (12)	0.46999 (10)	0.0528 (4)	
N1A	0.7964 (2)	−0.14877 (13)	0.09172 (10)	0.0604 (5)	
H1AA	0.7847	−0.1934	0.0445	0.072*	
C3D	0.7090 (2)	0.70904 (15)	0.37417 (11)	0.0507 (5)	
C3A	0.7648 (2)	−0.17061 (15)	0.15602 (12)	0.0541 (5)	
N3B	0.8723 (2)	−0.42118 (14)	−0.30377 (11)	0.0618 (5)	
C5A	0.66625 (19)	−0.48223 (14)	−0.03193 (12)	0.0493 (5)	
C4A	0.6790 (2)	−0.41118 (15)	0.05027 (13)	0.0512 (5)	
H4AA	0.6608	−0.4286	0.0948	0.061*	
C5D	0.8371 (2)	1.01651 (15)	0.56714 (12)	0.0517 (5)	
C4D	0.8144 (2)	0.94679 (15)	0.48379 (12)	0.0534 (5)	
H4DA	0.8388	0.9642	0.4401	0.064*	
N3C	0.5771 (2)	−0.04440 (14)	−0.17236 (11)	0.0680 (5)	
N2B	0.8282 (2)	−0.34282 (14)	−0.25800 (10)	0.0663 (5)	
H2BA	0.7763	−0.3203	−0.2807	0.080*	
N1B	0.9511 (2)	−0.34109 (14)	−0.14897 (11)	0.0666 (5)	
H1BA	0.9764	−0.3872	−0.1845	0.080*	
C3B	0.8681 (2)	−0.30171 (16)	−0.17630 (13)	0.0608 (6)	
C6A	0.6774 (2)	−0.45969 (16)	−0.10174 (13)	0.0610 (6)	
H6AA	0.6934	−0.3976	−0.0963	0.073*	
C8D	0.8865 (2)	1.15085 (16)	0.72642 (13)	0.0573 (5)	
C10D	0.8050 (2)	0.99256 (16)	0.63471 (13)	0.0583 (5)	
H10A	0.7679	0.9313	0.6269	0.070*	
N2C	0.6029 (3)	−0.12828 (16)	−0.22144 (11)	0.0800 (7)	
H2CA	0.6489	−0.1564	−0.2009	0.096*	
C8B	0.9502 (2)	−0.69882 (16)	−0.54091 (14)	0.0610 (5)	
C6D	0.8938 (2)	1.10765 (16)	0.58141 (14)	0.0598 (5)	
H6DA	0.9158	1.1247	0.5372	0.072*	
C8C	0.5907 (2)	0.24294 (16)	0.08527 (14)	0.0609 (5)	
C8A	0.6428 (2)	−0.62037 (17)	−0.18858 (14)	0.0635 (6)	
C5B	0.8749 (2)	−0.53968 (16)	−0.43370 (13)	0.0573 (5)	
C9A	0.6289 (2)	−0.64442 (17)	−0.12059 (16)	0.0666 (6)	
H9AA	0.6117	−0.7068	−0.1268	0.080*	
C7B	0.8681 (3)	−0.65617 (18)	−0.56913 (13)	0.0729 (7)	
H7BA	0.8373	−0.6800	−0.6245	0.087*	
C10A	0.6406 (2)	−0.57573 (16)	−0.04307 (14)	0.0591 (5)	
H10B	0.6311	−0.5927	0.0024	0.071*	
C9D	0.8283 (2)	1.05956 (17)	0.71286 (13)	0.0632 (6)	
H9DA	0.8046	1.0433	0.7572	0.076*	
C7A	0.6651 (3)	−0.52784 (19)	−0.17804 (14)	0.0705 (7)	
H7AA	0.6721	−0.5113	−0.2238	0.085*	
C4B	0.8349 (3)	−0.45683 (17)	−0.37994 (13)	0.0646 (6)	
H4BA	0.7803	−0.4292	−0.4019	0.078*	
C10B	0.9567 (2)	−0.58410 (18)	−0.40533 (13)	0.0650 (6)	
H10C	0.9866	−0.5609	−0.3499	0.078*	
C2D	0.6381 (3)	0.59073 (18)	0.43388 (14)	0.0745 (7)	
H2DB	0.6588	0.5894	0.4876	0.089*	
H2DC	0.6886	0.5522	0.3957	0.089*	
C3C	0.5568 (3)	−0.16705 (18)	−0.30147 (14)	0.0713 (7)	
C6C	0.5343 (3)	0.13004 (18)	−0.05242 (15)	0.0656 (6)	
H6CA	0.4886	0.1116	−0.1038	0.079*	
C9B	0.9939 (3)	−0.66138 (19)	−0.45749 (15)	0.0693 (6)	
H9BA	1.0496	−0.6896	−0.4370	0.083*	
C7D	0.9188 (2)	1.17422 (16)	0.66004 (14)	0.0628 (6)	
H7DA	0.9577	1.2352	0.6681	0.075*	
C4C	0.6275 (3)	−0.0129 (2)	−0.09849 (15)	0.0810 (8)	
H4CA	0.6772	−0.0480	−0.0823	0.097*	
C5C	0.6116 (3)	0.07495 (17)	−0.03801 (13)	0.0659 (6)	
C2A	0.8492 (3)	−0.05562 (18)	0.09475 (15)	0.0809 (9)	
H2AB	0.8181	−0.0110	0.1408	0.097*	
H2AC	0.8168	−0.0496	0.0450	0.097*	
N1C	0.4853 (3)	−0.12191 (17)	−0.32778 (13)	0.0853 (7)	
H1CA	0.4686	−0.0718	−0.2926	0.102*	
C6B	0.8302 (3)	−0.57779 (18)	−0.51630 (14)	0.0746 (7)	
H6BA	0.7735	−0.5502	−0.5367	0.089*	
C7C	0.5234 (3)	0.21250 (18)	0.00832 (16)	0.0709 (7)	
H7CA	0.4696	0.2484	−0.0027	0.085*	
C10C	0.6784 (4)	0.1063 (2)	0.03940 (17)	0.1071 (13)	
H10D	0.7324	0.0706	0.0507	0.129*	
C9C	0.6676 (4)	0.1891 (2)	0.10045 (16)	0.0900 (10)	
H9CA	0.7132	0.2078	0.1520	0.108*	
C2B	1.0029 (3)	−0.3124 (2)	−0.06345 (15)	0.0783 (7)	
H2BB	0.9320	−0.3077	−0.0288	0.094*	
H2BC	1.0604	−0.2517	−0.0462	0.094*	
C11D	0.9757 (3)	1.3390 (2)	0.82323 (17)	0.0862 (9)	
H11A	0.9925	1.3859	0.8775	0.129*	
H11B	0.9135	1.3537	0.7913	0.129*	
H11C	1.0553	1.3367	0.7976	0.129*	
C1B	1.0759 (4)	−0.3812 (3)	−0.05415 (18)	0.1076 (12)	
H1BB	1.1090	−0.3622	0.0023	0.161*	
H1BC	1.1470	−0.3848	−0.0876	0.161*	
H1BD	1.0187	−0.4412	−0.0711	0.161*	
C11A	0.5706 (3)	−0.8097 (2)	−0.2859 (2)	0.1097 (12)	
H11D	0.5645	−0.8573	−0.3395	0.165*	
H11E	0.4854	−0.8092	−0.2675	0.165*	
H11F	0.6258	−0.8221	−0.2482	0.165*	
C1A	0.9928 (4)	−0.0328 (2)	0.1032 (2)	0.1091 (12)	
H1AB	1.0221	0.0298	0.1060	0.164*	
H1AC	1.0243	−0.0750	0.0566	0.164*	
H1AD	1.0256	−0.0384	0.1525	0.164*	
C11C	0.6779 (4)	0.3679 (2)	0.24419 (17)	0.0959 (10)	
H11G	0.6743	0.4263	0.2867	0.144*	
H11H	0.7655	0.3683	0.2299	0.144*	
H11I	0.6503	0.3183	0.2635	0.144*	
C1D	0.4976 (4)	0.5507 (2)	0.4078 (2)	0.1096 (12)	
H1DB	0.4785	0.4877	0.4054	0.164*	
H1DC	0.4764	0.5516	0.3546	0.164*	
H1DD	0.4470	0.5869	0.4466	0.164*	
C11B	0.9299 (4)	−0.8277 (2)	−0.70075 (18)	0.1145 (13)	
H11J	0.9563	−0.8820	−0.7376	0.172*	
H11K	0.9548	−0.7770	−0.7190	0.172*	
H11L	0.8371	−0.8407	−0.6999	0.172*	
C2C	0.4309 (5)	−0.1508 (3)	−0.4138 (2)	0.1359 (18)	
H2CB	0.4887	−0.1851	−0.4491	0.163*	
H2CC	0.3478	−0.1933	−0.4218	0.163*	
C1C	0.4128 (6)	−0.0818 (4)	−0.4382 (3)	0.172 (2)	
H1CB	0.3779	−0.1075	−0.4948	0.258*	
H1CC	0.4945	−0.0398	−0.4319	0.258*	
H1CD	0.3529	−0.0487	−0.4053	0.258*	

### Atomic displacement parameters (Å^2^)

**Table:**

	*U*^11^	*U*^22^	*U*^33^	*U*^12^	*U*^13^	*U*^23^
S1D	0.0967 (5)	0.0526 (3)	0.0385 (3)	0.0130 (3)	0.0152 (3)	0.0077 (2)
S2A	0.1070 (5)	0.0539 (3)	0.0419 (3)	0.0123 (3)	0.0111 (3)	0.0065 (2)
S1B	0.1203 (6)	0.0505 (3)	0.0483 (3)	0.0269 (3)	0.0128 (3)	0.0075 (2)
S2D	0.0965 (5)	0.0675 (4)	0.0514 (3)	0.0182 (4)	0.0026 (3)	−0.0028 (3)
S1C	0.1435 (7)	0.0710 (5)	0.0468 (3)	0.0242 (5)	0.0222 (4)	0.0065 (3)
S2C	0.1155 (6)	0.0633 (4)	0.0799 (4)	0.0435 (4)	0.0096 (4)	0.0041 (3)
S1A	0.1066 (6)	0.0885 (6)	0.0643 (4)	0.0383 (5)	0.0037 (4)	−0.0109 (4)
S2B	0.1272 (7)	0.0838 (5)	0.0892 (5)	0.0574 (5)	0.0367 (5)	0.0136 (4)
N3A	0.0729 (11)	0.0503 (10)	0.0397 (8)	0.0053 (9)	0.0075 (8)	0.0106 (7)
N3D	0.0909 (13)	0.0461 (10)	0.0395 (8)	0.0157 (9)	0.0130 (9)	0.0101 (7)
N2A	0.0592 (10)	0.0494 (10)	0.0410 (8)	0.0119 (8)	0.0037 (7)	0.0082 (7)
N2D	0.0743 (11)	0.0474 (10)	0.0395 (8)	0.0061 (8)	0.0095 (8)	0.0102 (7)
N1D	0.0615 (10)	0.0479 (10)	0.0411 (8)	0.0110 (8)	0.0028 (8)	0.0075 (7)
N1A	0.0905 (13)	0.0443 (10)	0.0422 (9)	0.0149 (9)	0.0042 (9)	0.0108 (7)
C3D	0.0574 (11)	0.0493 (11)	0.0402 (9)	0.0110 (9)	0.0043 (9)	0.0105 (8)
C3A	0.0659 (12)	0.0485 (11)	0.0436 (10)	0.0150 (10)	0.0040 (9)	0.0104 (9)
N3B	0.0800 (12)	0.0551 (11)	0.0453 (9)	0.0186 (9)	0.0140 (9)	0.0100 (8)
C5A	0.0441 (9)	0.0489 (11)	0.0492 (10)	0.0115 (8)	0.0030 (8)	0.0106 (9)
C4A	0.0556 (11)	0.0486 (12)	0.0478 (10)	0.0104 (9)	0.0065 (9)	0.0161 (9)
C5D	0.0540 (11)	0.0493 (11)	0.0469 (10)	0.0130 (9)	0.0074 (9)	0.0106 (9)
C4D	0.0621 (12)	0.0494 (12)	0.0451 (10)	0.0117 (10)	0.0070 (9)	0.0130 (9)
N3C	0.0942 (14)	0.0589 (12)	0.0491 (10)	0.0246 (11)	0.0133 (10)	0.0130 (9)
N2B	0.0976 (14)	0.0554 (11)	0.0430 (9)	0.0286 (10)	0.0108 (10)	0.0082 (8)
N1B	0.0814 (13)	0.0629 (12)	0.0460 (9)	0.0213 (10)	0.0070 (9)	0.0057 (8)
C3B	0.0774 (14)	0.0512 (12)	0.0452 (11)	0.0066 (11)	0.0152 (10)	0.0101 (9)
C6A	0.0747 (14)	0.0515 (13)	0.0503 (11)	0.0073 (11)	0.0003 (11)	0.0145 (10)
C8D	0.0573 (11)	0.0520 (12)	0.0506 (11)	0.0149 (10)	0.0039 (10)	0.0034 (9)
C10D	0.0697 (13)	0.0490 (12)	0.0479 (11)	0.0070 (10)	0.0019 (10)	0.0110 (9)
N2C	0.1209 (18)	0.0689 (14)	0.0436 (10)	0.0397 (13)	0.0111 (11)	0.0033 (9)
C8B	0.0715 (13)	0.0535 (13)	0.0585 (12)	0.0197 (11)	0.0223 (11)	0.0169 (10)
C6D	0.0714 (14)	0.0493 (12)	0.0526 (11)	0.0095 (10)	0.0107 (10)	0.0129 (9)
C8C	0.0732 (14)	0.0511 (12)	0.0585 (12)	0.0226 (11)	0.0109 (11)	0.0154 (10)
C8A	0.0585 (12)	0.0613 (14)	0.0571 (12)	0.0210 (11)	0.0045 (10)	0.0019 (11)
C5B	0.0706 (13)	0.0556 (13)	0.0444 (10)	0.0187 (11)	0.0105 (10)	0.0139 (9)
C9A	0.0701 (14)	0.0455 (12)	0.0763 (15)	0.0192 (11)	0.0077 (12)	0.0097 (11)
C7B	0.106 (2)	0.0682 (16)	0.0422 (11)	0.0349 (15)	0.0066 (12)	0.0091 (11)
C10A	0.0672 (13)	0.0519 (12)	0.0585 (12)	0.0164 (10)	0.0078 (11)	0.0186 (10)
C9D	0.0747 (14)	0.0642 (14)	0.0463 (11)	0.0131 (12)	0.0064 (11)	0.0159 (10)
C7A	0.0849 (16)	0.0728 (17)	0.0473 (11)	0.0153 (13)	0.0049 (12)	0.0150 (11)
C4B	0.0866 (16)	0.0567 (13)	0.0472 (11)	0.0237 (12)	0.0081 (11)	0.0107 (10)
C10B	0.0782 (15)	0.0688 (15)	0.0458 (11)	0.0223 (12)	0.0016 (11)	0.0156 (10)
C2D	0.121 (2)	0.0592 (14)	0.0466 (11)	0.0271 (15)	0.0150 (13)	0.0188 (10)
C3C	0.0975 (18)	0.0653 (15)	0.0440 (11)	0.0066 (14)	0.0162 (12)	0.0161 (11)
C6C	0.0747 (14)	0.0615 (14)	0.0585 (13)	0.0219 (12)	−0.0009 (11)	0.0165 (11)
C9B	0.0745 (15)	0.0723 (16)	0.0648 (14)	0.0324 (13)	0.0087 (12)	0.0214 (12)
C7D	0.0689 (14)	0.0452 (12)	0.0634 (13)	0.0093 (10)	0.0091 (11)	0.0082 (10)
C4C	0.120 (2)	0.0703 (17)	0.0514 (13)	0.0495 (16)	−0.0003 (14)	0.0073 (11)
C5C	0.0911 (17)	0.0586 (14)	0.0498 (11)	0.0332 (13)	0.0062 (12)	0.0137 (10)
C2A	0.150 (3)	0.0504 (14)	0.0482 (12)	0.0322 (16)	0.0097 (15)	0.0191 (10)
N1C	0.1164 (19)	0.0747 (15)	0.0538 (11)	0.0089 (14)	−0.0073 (12)	0.0169 (11)
C6B	0.110 (2)	0.0676 (16)	0.0466 (11)	0.0409 (15)	0.0020 (13)	0.0111 (11)
C7C	0.0818 (16)	0.0571 (14)	0.0741 (15)	0.0285 (12)	0.0001 (13)	0.0183 (12)
C10C	0.174 (3)	0.086 (2)	0.0610 (15)	0.083 (2)	−0.0183 (19)	0.0023 (14)
C9C	0.139 (3)	0.0724 (17)	0.0543 (13)	0.0541 (18)	−0.0147 (16)	0.0044 (12)
C2B	0.0872 (17)	0.0840 (19)	0.0493 (12)	0.0206 (15)	−0.0004 (12)	0.0069 (12)
C11D	0.0785 (17)	0.0711 (17)	0.0765 (17)	0.0053 (14)	0.0112 (14)	−0.0065 (13)
C1B	0.125 (3)	0.136 (3)	0.0639 (16)	0.062 (2)	0.0024 (18)	0.0238 (18)
C11A	0.0859 (19)	0.083 (2)	0.114 (3)	0.0141 (17)	0.0107 (19)	−0.0176 (18)
C1A	0.133 (3)	0.067 (2)	0.118 (3)	−0.0126 (19)	0.008 (2)	0.0404 (18)
C11C	0.133 (3)	0.0739 (19)	0.0641 (16)	0.0293 (18)	0.0092 (17)	0.0015 (13)
C1D	0.124 (3)	0.082 (2)	0.112 (3)	−0.014 (2)	0.011 (2)	0.040 (2)
C11B	0.177 (4)	0.074 (2)	0.0721 (18)	0.026 (2)	0.041 (2)	0.0006 (15)
C2C	0.217 (5)	0.098 (3)	0.074 (2)	0.007 (3)	−0.044 (3)	0.0247 (19)
C1C	0.240 (6)	0.159 (5)	0.125 (3)	0.067 (4)	−0.062 (4)	0.054 (3)

### Geometric parameters (Å, °)

**Table:**

S1D—C3D	1.681 (2)	C9A—C10A	1.385 (3)
S2A—C3A	1.684 (2)	C9A—H9AA	0.9300
S1B—C3B	1.670 (3)	C7B—C6B	1.384 (3)
S2D—C8D	1.758 (2)	C7B—H7BA	0.9300
S2D—C11D	1.780 (3)	C10A—H10B	0.9300
S1C—C3C	1.688 (3)	C9D—H9DA	0.9300
S2C—C8C	1.758 (2)	C7A—H7AA	0.9300
S2C—C11C	1.770 (3)	C4B—H4BA	0.9300
S1A—C8A	1.767 (2)	C10B—C9B	1.364 (3)
S1A—C11A	1.773 (4)	C10B—H10C	0.9300
S2B—C8B	1.749 (2)	C2D—C1D	1.483 (5)
S2B—C11B	1.771 (4)	C2D—H2DB	0.9700
N3A—C3A	1.350 (3)	C2D—H2DC	0.9700
N3A—N2A	1.372 (2)	C3C—N1C	1.300 (4)
N3A—H3AA	0.8600	C6C—C5C	1.369 (3)
N3D—C3D	1.329 (3)	C6C—C7C	1.378 (3)
N3D—C2D	1.450 (3)	C6C—H6CA	0.9300
N3D—H3DA	0.8600	C9B—H9BA	0.9300
N2A—C4A	1.278 (3)	C7D—H7DA	0.9300
N2D—C3D	1.353 (3)	C4C—C5C	1.451 (3)
N2D—N1D	1.366 (2)	C4C—H4CA	0.9300
N2D—H2DA	0.8600	C5C—C10C	1.385 (4)
N1D—C4D	1.273 (3)	C2A—C1A	1.476 (5)
N1A—C3A	1.326 (3)	C2A—H2AB	0.9700
N1A—C2A	1.451 (3)	C2A—H2AC	0.9700
N1A—H1AA	0.8600	N1C—C2C	1.474 (4)
N3B—C4B	1.267 (3)	N1C—H1CA	0.8600
N3B—N2B	1.377 (3)	C6B—H6BA	0.9300
C5A—C10A	1.386 (3)	C7C—H7CA	0.9300
C5A—C6A	1.394 (3)	C10C—C9C	1.383 (4)
C5A—C4A	1.457 (3)	C10C—H10D	0.9300
C4A—H4AA	0.9300	C9C—H9CA	0.9300
C5D—C6D	1.378 (3)	C2B—C1B	1.485 (4)
C5D—C10D	1.399 (3)	C2B—H2BB	0.9700
C5D—C4D	1.460 (3)	C2B—H2BC	0.9700
C4D—H4DA	0.9300	C11D—H11A	0.9600
N3C—C4C	1.268 (3)	C11D—H11B	0.9600
N3C—N2C	1.372 (3)	C11D—H11C	0.9600
N2B—C3B	1.360 (3)	C1B—H1BB	0.9600
N2B—H2BA	0.8600	C1B—H1BC	0.9600
N1B—C3B	1.326 (3)	C1B—H1BD	0.9600
N1B—C2B	1.459 (3)	C11A—H11D	0.9600
N1B—H1BA	0.8600	C11A—H11E	0.9600
C6A—C7A	1.366 (3)	C11A—H11F	0.9600
C6A—H6AA	0.9300	C1A—H1AB	0.9600
C8D—C7D	1.375 (3)	C1A—H1AC	0.9600
C8D—C9D	1.386 (3)	C1A—H1AD	0.9600
C10D—C9D	1.378 (3)	C11C—H11G	0.9600
C10D—H10A	0.9300	C11C—H11H	0.9600
N2C—C3C	1.347 (3)	C11C—H11I	0.9600
N2C—H2CA	0.8600	C1D—H1DB	0.9600
C8B—C7B	1.364 (3)	C1D—H1DC	0.9600
C8B—C9B	1.394 (3)	C1D—H1DD	0.9600
C6D—C7D	1.382 (3)	C11B—H11J	0.9600
C6D—H6DA	0.9300	C11B—H11K	0.9600
C8C—C9C	1.357 (4)	C11B—H11L	0.9600
C8C—C7C	1.382 (3)	C2C—C1C	1.344 (5)
C8A—C7A	1.375 (4)	C2C—H2CB	0.9700
C8A—C9A	1.379 (4)	C2C—H2CC	0.9700
C5B—C6B	1.384 (3)	C1C—H1CB	0.9600
C5B—C10B	1.384 (3)	C1C—H1CC	0.9600
C5B—C4B	1.450 (3)	C1C—H1CD	0.9600
			
C8D—S2D—C11D	104.40 (13)	H2DB—C2D—H2DC	107.8
C8C—S2C—C11C	104.08 (13)	N1C—C3C—N2C	116.4 (2)
C8A—S1A—C11A	104.33 (15)	N1C—C3C—S1C	124.9 (2)
C8B—S2B—C11B	104.45 (16)	N2C—C3C—S1C	118.7 (2)
C3A—N3A—N2A	119.28 (17)	C5C—C6C—C7C	120.8 (2)
C3A—N3A—H3AA	120.4	C5C—C6C—H6CA	119.6
N2A—N3A—H3AA	120.4	C7C—C6C—H6CA	119.6
C3D—N3D—C2D	124.72 (18)	C10B—C9B—C8B	121.3 (2)
C3D—N3D—H3DA	117.6	C10B—C9B—H9BA	119.4
C2D—N3D—H3DA	117.6	C8B—C9B—H9BA	119.4
C4A—N2A—N3A	117.34 (18)	C8D—C7D—C6D	120.4 (2)
C3D—N2D—N1D	119.31 (17)	C8D—C7D—H7DA	119.8
C3D—N2D—H2DA	120.3	C6D—C7D—H7DA	119.8
N1D—N2D—H2DA	120.3	N3C—C4C—C5C	123.3 (2)
C4D—N1D—N2D	117.00 (18)	N3C—C4C—H4CA	118.3
C3A—N1A—C2A	125.21 (19)	C5C—C4C—H4CA	118.3
C3A—N1A—H1AA	117.4	C6C—C5C—C10C	117.1 (2)
C2A—N1A—H1AA	117.4	C6C—C5C—C4C	124.1 (2)
N3D—C3D—N2D	116.24 (18)	C10C—C5C—C4C	118.8 (2)
N3D—C3D—S1D	123.83 (17)	N1A—C2A—C1A	113.2 (2)
N2D—C3D—S1D	119.84 (15)	N1A—C2A—H2AB	108.9
N1A—C3A—N3A	115.98 (18)	C1A—C2A—H2AB	108.9
N1A—C3A—S2A	124.11 (17)	N1A—C2A—H2AC	108.9
N3A—C3A—S2A	119.91 (16)	C1A—C2A—H2AC	108.9
C4B—N3B—N2B	117.1 (2)	H2AB—C2A—H2AC	107.8
C10A—C5A—C6A	117.6 (2)	C3C—N1C—C2C	124.4 (3)
C10A—C5A—C4A	120.3 (2)	C3C—N1C—H1CA	117.8
C6A—C5A—C4A	122.0 (2)	C2C—N1C—H1CA	117.8
N2A—C4A—C5A	120.58 (19)	C5B—C6B—C7B	121.3 (2)
N2A—C4A—H4AA	119.7	C5B—C6B—H6BA	119.3
C5A—C4A—H4AA	119.7	C7B—C6B—H6BA	119.3
C6D—C5D—C10D	118.2 (2)	C6C—C7C—C8C	121.4 (2)
C6D—C5D—C4D	120.6 (2)	C6C—C7C—H7CA	119.3
C10D—C5D—C4D	121.2 (2)	C8C—C7C—H7CA	119.3
N1D—C4D—C5D	121.0 (2)	C9C—C10C—C5C	122.2 (3)
N1D—C4D—H4DA	119.5	C9C—C10C—H10D	118.9
C5D—C4D—H4DA	119.5	C5C—C10C—H10D	118.9
C4C—N3C—N2C	115.7 (2)	C8C—C9C—C10C	120.1 (2)
C3B—N2B—N3B	118.8 (2)	C8C—C9C—H9CA	120.0
C3B—N2B—H2BA	120.6	C10C—C9C—H9CA	120.0
N3B—N2B—H2BA	120.6	N1B—C2B—C1B	110.0 (2)
C3B—N1B—C2B	125.2 (2)	N1B—C2B—H2BB	109.7
C3B—N1B—H1BA	117.4	C1B—C2B—H2BB	109.7
C2B—N1B—H1BA	117.4	N1B—C2B—H2BC	109.7
N1B—C3B—N2B	115.0 (2)	C1B—C2B—H2BC	109.7
N1B—C3B—S1B	124.81 (17)	H2BB—C2B—H2BC	108.2
N2B—C3B—S1B	120.1 (2)	S2D—C11D—H11A	109.5
C7A—C6A—C5A	120.7 (2)	S2D—C11D—H11B	109.5
C7A—C6A—H6AA	119.6	H11A—C11D—H11B	109.5
C5A—C6A—H6AA	119.6	S2D—C11D—H11C	109.5
C7D—C8D—C9D	118.8 (2)	H11A—C11D—H11C	109.5
C7D—C8D—S2D	124.82 (18)	H11B—C11D—H11C	109.5
C9D—C8D—S2D	116.42 (18)	C2B—C1B—H1BB	109.5
C9D—C10D—C5D	120.1 (2)	C2B—C1B—H1BC	109.5
C9D—C10D—H10A	119.9	H1BB—C1B—H1BC	109.5
C5D—C10D—H10A	119.9	C2B—C1B—H1BD	109.5
C3C—N2C—N3C	120.6 (2)	H1BB—C1B—H1BD	109.5
C3C—N2C—H2CA	119.7	H1BC—C1B—H1BD	109.5
N3C—N2C—H2CA	119.7	S1A—C11A—H11D	109.5
C7B—C8B—C9B	118.1 (2)	S1A—C11A—H11E	109.5
C7B—C8B—S2B	125.57 (19)	H11D—C11A—H11E	109.5
C9B—C8B—S2B	116.4 (2)	S1A—C11A—H11F	109.5
C5D—C6D—C7D	121.4 (2)	H11D—C11A—H11F	109.5
C5D—C6D—H6DA	119.3	H11E—C11A—H11F	109.5
C7D—C6D—H6DA	119.3	C2A—C1A—H1AB	109.5
C9C—C8C—C7C	118.4 (2)	C2A—C1A—H1AC	109.5
C9C—C8C—S2C	124.19 (19)	H1AB—C1A—H1AC	109.5
C7C—C8C—S2C	117.39 (18)	C2A—C1A—H1AD	109.5
C7A—C8A—C9A	118.9 (2)	H1AB—C1A—H1AD	109.5
C7A—C8A—S1A	116.1 (2)	H1AC—C1A—H1AD	109.5
C9A—C8A—S1A	125.0 (2)	S2C—C11C—H11G	109.5
C6B—C5B—C10B	117.6 (2)	S2C—C11C—H11H	109.5
C6B—C5B—C4B	120.2 (2)	H11G—C11C—H11H	109.5
C10B—C5B—C4B	122.3 (2)	S2C—C11C—H11I	109.5
C8A—C9A—C10A	120.0 (2)	H11G—C11C—H11I	109.5
C8A—C9A—H9AA	120.0	H11H—C11C—H11I	109.5
C10A—C9A—H9AA	120.0	C2D—C1D—H1DB	109.5
C8B—C7B—C6B	120.7 (2)	C2D—C1D—H1DC	109.5
C8B—C7B—H7BA	119.6	H1DB—C1D—H1DC	109.5
C6B—C7B—H7BA	119.6	C2D—C1D—H1DD	109.5
C9A—C10A—C5A	121.3 (2)	H1DB—C1D—H1DD	109.5
C9A—C10A—H10B	119.3	H1DC—C1D—H1DD	109.5
C5A—C10A—H10B	119.3	S2B—C11B—H11J	109.5
C10D—C9D—C8D	121.1 (2)	S2B—C11B—H11K	109.5
C10D—C9D—H9DA	119.5	H11J—C11B—H11K	109.5
C8D—C9D—H9DA	119.5	S2B—C11B—H11L	109.5
C6A—C7A—C8A	121.4 (2)	H11J—C11B—H11L	109.5
C6A—C7A—H7AA	119.3	H11K—C11B—H11L	109.5
C8A—C7A—H7AA	119.3	C1C—C2C—N1C	115.8 (4)
N3B—C4B—C5B	121.7 (2)	C1C—C2C—H2CB	108.3
N3B—C4B—H4BA	119.2	N1C—C2C—H2CB	108.3
C5B—C4B—H4BA	119.2	C1C—C2C—H2CC	108.3
C9B—C10B—C5B	121.0 (2)	N1C—C2C—H2CC	108.3
C9B—C10B—H10C	119.5	H2CB—C2C—H2CC	107.4
C5B—C10B—H10C	119.5	C2C—C1C—H1CB	109.5
N3D—C2D—C1D	113.1 (3)	C2C—C1C—H1CC	109.5
N3D—C2D—H2DB	109.0	H1CB—C1C—H1CC	109.5
C1D—C2D—H2DB	109.0	C2C—C1C—H1CD	109.5
N3D—C2D—H2DC	109.0	H1CB—C1C—H1CD	109.5
C1D—C2D—H2DC	109.0	H1CC—C1C—H1CD	109.5

### Hydrogen-bond geometry (Å, °)

**Table:**

*D*—H···*A*	*D*—H	H···*A*	*D*···*A*	*D*—H···*A*
N1A—H1AA···N2A	0.86	2.20	2.602 (3)	108
N1B—H1BA···N3B	0.86	2.17	2.585 (3)	109
N1C—H1CA···N3C	0.86	2.23	2.624 (3)	108
N3D—H3DA···N1D	0.86	2.22	2.610 (3)	107
N3A—H3AA···S1D^i^	0.86	2.59	3.398 (2)	156
N2D—H2DA···S2A^ii^	0.86	2.57	3.402 (2)	163
N1A—H1AA···S1B	0.86	2.81	3.4798 (19)	136
N2B—H2BA···S1C	0.86	2.72	3.579 (2)	174
N2C—H2CA···S1B	0.86	2.64	3.487 (3)	168

Symmetry codes: (i) *x*, *y*−1, *z*; (ii) *x*, *y*+1, *z*.
